# Bridge Over Troubled Waters? Certified Recovery Specialist Support and Community Reentry: A Pilot Study

**DOI:** 10.1177/0306624X251322824

**Published:** 2025-02-24

**Authors:** Derek A. Kreager, Yiwen Zhang, Deirdre O’Sullivan, Gary Zajac, Kristofer Bret Bucklen

**Affiliations:** 1 Pennsylvania State University, University Park, PA, USA; 2 Pennsylvania Department of Corrections, Mechanicsburg, PA, USA

**Keywords:** reentry, peer support, substance use disorder, families, recovery capital

## Abstract

Peer support services, including certified recovery specialists (CRSs), have been of increasing interest to treatment scholars. However, if and how such services assist justice-involved individuals with substance use disorders during community reentry is less understood. This pilot study provided CRS services to both reentrants and their family members during the transition from close custody confinement to community supervision, a perilous period in which risks of relapse and rearrest peak. Post-intervention interviews with nine of fifteen recruited reentrants and four of five recruited family members in central Pennsylvania were analyzed using iterative thematic coding. Participants perceived CRS services as essential for the reentry and recovery process. Interviewees identified CRS’s lived experience, advocacy, availability, empathy, and family outreach as key mechanisms of recovery success. These results highlight the importance of CRS services for recovery capital during the community reentry transition.

The United States leads the world in the total number of people incarcerated (World Prison Brief, 2021). Commonly overlooked, however, is that the vast majority of those incarcerated will eventually be released, with more than 600,000 incarcerated Americans returning to their communities each year (Carson & Golinelli, 2014; Petersilia, 2003). Distressingly, over half of these reentrants will be rearrested within the first year of release and 76% within 5 years (Durose et al., 2014). Furthermore, about two thirds of the U.S. prison population are diagnosed with substance use disorders (SUD) (Mumola & Karberg, 2006) and about one third of these report relapse within ten months of release (Gunnison & Helfgott, 2013; Mallik-Kane & Visher, 2008). Relapse, or the continuation of drug use, is a parole violation resulting in reincarceration for many reentrants. Additionally, the risk of fatal overdose peaks in the 2 weeks after community reentry (Binswanger et al., 2013).

The high prevalence of substance use disorders among incarcerated populations and high relapse and overdose rates have motivated alternative sentencing programs (e.g., drug court) and improvements to prison-based substance use treatment (e.g., medication-assisted treatment, therapeutic communities, cognitive behavioral therapy). Less attention, however, has been directed at preparing for reentry and continuity of care after release. Reentrants face many obstacles during reentry: employment, housing, health care, social reintegration, discrimination and stigma (Mallik-Kane & Visher, 2008; Visher & Travis, 2003). Without a smooth transition and continuity of care, reentrants with SUD are at high risk of relapse, rearrest, overdose and related fatality immediately following institutional release (Binswanger et al., 2013; Lim et al., 2012; Merrall et al., 2010). Identifying recovery-specific supports and services to assist SUD reentrants is thus a crucial task for correctional researchers and policy makers. For instance, the importance of developing and implementing policies and practices that facilitate a “warm hand-off” between correctional agencies and community service providers was highlighted by the Substance Abuse and Mental Health Services Administration (SAMHSA, 2017) in their guidelines for successful transition and recovery of people with mental or substance use disorders released from jail and prison.

Within the SUD treatment literature, the concept of Recovery Capital (RC) has gained traction for its connection to sustained recovery efforts and treatment outcomes (Laudet & White, 2008; Obekpa et al., 2023; Sánchez et al., 2020). RC borrows from the construct of social capital (Bourdieu, 1985; Coleman, 1988), which explains how individuals and families benefit from the people in their social networks (Portes, 1998). Social capital, in turn, is closely tied to economic capital in the sense that social ties to people with education, access to resources, jobs, and other sources of wealth and prestige enhance life outcomes. In other words, connections to the “right” people lead to social capital, whereas connections to those with limited education, wealth, or resources do not produce social capital (Portes, 1998) or promote positive “turning points” in individuals’ lives (Sampson & Laub, 1993). Similarly, RC is enhanced when an individual in recovery is connected to certain kinds of people and resources. RC tends to be a network of supports including general social supports and recovery-specific supports (Best & Hennessy, 2022; Hennessy, 2017). RC is often described as an ecological construct that includes micro- (e.g., individual or dyadic) and macro-level (e.g., agency, family, or community) domains, with each domain interacting with others to either strengthen or weaken the overall fabric of RC. Recovery allies and treatment providers aim to increase access to RC with the goal of more RC contributing to sustained recovery over time (Cloud & Granfield, 2008). One of the most consistently cited sources of RC includes positive social supports, in the forms of peers, professionals, and family members (Hennessy, 2017; Laudet & White, 2008). Certified Recovery Specialists (CRSs) are potential sources of RC that have not received extensive research investigation. The current study helps fill this gap by assessing how inclusion of a CRS during community re-entry can facilitate successful reintegration for incarcerated individuals with SUDs.

## Literature Review

Certified recovery specialists (also known as peer recovery specialists, peer support specialists, or recovery coaches) are promising sources of RC (Bassuk et al., 2016; Eddie et al., 2019; Tolan et al., 2013). Successful in their own recovery process, CRSs are trained and certified (typically by state agencies) individuals who provide social support and non-clinical services to peers in recovery from SUD. Unlike other clinical or SUD counseling positions, attainment of higher education is not required for CRSs, but specific training in recovery support education is compulsory, with additional requirements in ethics, substance use, trauma, suicide prevention, among other topics depending on the state (Beck et al., 2018). One key requirement is that CRSs must testify to their own recovery, with different states requiring varying amounts of time in recovery (Beck et al., 2018). CRS assistance does not replace, but rather complements and augments treatment, outreach, engagement, and other strategies and interventions that aid long-term recovery. Studies have documented a wide range of services provided by CRSs for justice-involved individuals with substance use disorders, including connections to local resources and services, transportation, individualized recovery plans, and guidance and motivational support drawing on their own lived experiences (Cos et al., 2020; Laudet & Humphreys, 2013; Tillson et al., 2022).

Although relatively new to behavioral health services, the promise of CRSs for recovery was highlighted in the landmark Surgeon General’s Report on Alcohol, Drugs, and Health (Office of the Surgeon General 2016), Substance Abuse and Mental Health Administration’s Evidence-Based Resource Guide (SAMSHA, 2023), and has received some empirical support from randomized controlled studies (Bernstein et al., 2005; O’Connell et al., 2020; Ray et al., 2021; Rowe et al., 2007; Tracy et al., 2011). Systematic and meta-analysis reviews show that peer recovery support services, in general, are associated with promising outcomes, such as reductions in substance use and rehospitalization, treatment program participation and engagement, and reduced risks of reoffending and comorbidities (e.g., hepatitis-C and HIV) (Bassuk et al., 2016; Eddie et al., 2019; Stack et al., 2022; Tolan et al., 2013). Several qualitative studies also point to the mechanisms connecting CRSs to positive client outcomes. For example, Kirk et al. (2025) found that individuals receiving CRS support during emergency room visits reported themes of (a) mutual respect and trust, (b) CRS adaptation to patients’ circumstances, and (c) personalized and comprehensive care as resulting in positive and impactful CRS interactions. Two other qualitative studies by Anvari et al. (2022) and Lennox et al. (2021) found that patients and providers reported peer-recovery specialists were important in removing stigma-related barriers in accessing treatment, advocating for patient services, and thus acting as bridges between the healthcare system and people who use drugs. As one employer stated of peer-recovery specialists (Lennox et al. [2021, p. 4]), “they see that there’s value in connecting with the system, but they also recognize that it isn’t easy, and that it can be very stigmatizing. . .So, I think that’s where the value comes in: it’s that bridge and buffer between a system that can actually do harm in some ways and also benefit.” Overall, both quantitative and qualitative studies support the integration of peer recovery support into community outreach programs, especially for marginalized and stigmatized populations (e.g., unhoused, having co-occurring mental health and substance use disorders, racial and ethnic minorities, and justice-involved individuals).

Indeed, CRSs are particularly attractive for justice-involved populations with SUD. The shared, relatable experiences of SUD and recovery often enable CRSs to easily bond with peers in recovery and earn their trust in comparison to institutional actors and medical professionals (Reingle Gonzalez et al., 2019). In addition, justice-involved individuals receiving peer support may perceive CRSs as role models whose experiences and paths to recovery and reentry are motivational and imitable (Schinkel & Whyte, 2012). Although sparse, results from recent empirical studies examining the inclusion of CRS services in criminal justice contexts suggest that peer support is associated with reductions in self-reported crime, rearrest, and reincarceration (Bauldry et al., 2009; Belenko et al., 2021; Bellamy et al., 2019; Cos et al., 2020). CRS services are also linked to better health outcomes for reentry and recovery populations, including improved self-efficacy, treatment adherence, and reduced substance use behaviors (Goldstein et al., 2009; Marlow et al., 2015; Ray et al., 2021; Tracy et al., 2011). Although promising, it should be noted that few of these studies focus directly on peer support, substance use, and reentry from jail or prison. Instead, most participants of these studies were from drug courts (Belenko et al., 2021), primary care settings (Cos et al., 2020), those with co-occurring mental illness and substance use jail participants (Bellamy et al., 2019; Goldstein et al., 2009), those without pre-existing substance use disorders (Marlow et al., 2015), and veterans (Tracy et al., 2011). Ray et al. (2021) provide the most relevant study for the current analysis. These authors implemented a pilot randomized controlled trial with state-certified peer recovery coaches randomly assigned to 100 participants released from incarceration and provided nonclinical services by the coaches. Ray et al. (2021) found that treated participants had improvements in treatment motivation, self-efficacy, and reduced substance use. We build on this research with a qualitative analysis of the mechanisms potentially underlying such peer recovery interventions.

Family members can be supportive, apathetic, or even iatrogenic for the recovery of reentrants with SUD (Comfort, 2016; Green et al., 2006; Mowen & Visher, 2016). In such variable circumstances, CRSs can take instrumental roles in understanding and reacting to reentrants’ family contexts, even prior to prison release. Specifically, CRSs can educate family members on the recovery and reentry process, sidestep negative family relationships, or even reignite weakened ties. A CRS intervention may thus generate beneficial spillover effects for family members, freeing them from service delivery roles that they are otherwise unqualified to provide while solidifying their role as critical social support for reentrants. Moreover, connecting CRSs and family members can strengthen the recovery capital (Cos et al., 2020; Victor et al., 2021) and social bonds (Hirschi, 1969) available to reentrants, which promote a supportive and positive community environment as well as criminal desistance.

An intervention that combines CRS services with a family-focused orientation, therefore, appears particularly attractive at reducing reentrants’ relapse and increasing their likelihood of reintegration success, particularly when viewed from the RC lens. To enhance the overall fabric of RC for any individual, it is important to broaden not only access to resources but enhance the interaction among these resources. In this case, the interaction of a reentrant, a CRS, and a caregiver or family member has more than additive value: it may have the potential for exponentially enhancing RC, a known predictor of recovery outcomes. However, we are unaware of any such program currently in existence. In addition, despite statistical associations with desirable outcomes shown in some studies, scholars have remarked that such results may be program dependent and that, because CRS services have just started to burgeon in the criminal justice settings and vary extensively, identifying underlying mechanisms for these associations is much needed before quantifying program design, implementation, and effectiveness. (Ray et al., 2021; Sells et al., 2020). To fill these gaps, this pilot study aimed to assess a CRS program that focused on the transition period from close custody confinement to community reentry, providing social support and resources to both reentrants and their family members, and to understand the mechanisms of “what works” through the views of the pilot participants.

## Method

### Study Design

This study consists of a pilot program targeting individuals sentenced to the Pennsylvania State Drug Treatment Program (SDTP; previously the State Intermediate Punishment program). SDTP is an alternative sanction for incarcerated individuals diagnosed with a SUD and consists of four levels of treatment over 24 months: 7 months of prison-based therapeutic community treatment, 2 months of community-based inpatient treatment, 6 months of community-based outpatient treatment, and the balance of the 24 months in community reintegration activities (e.g., educational or employment goals). Upon successful completion, SDTP participants are deemed to have served the entirety of their original sentence, including parole (for more information on the SDTP sentencing program, see https://www.cor.pa.gov/community-reentry/Pages/JRI2.aspx).

For this study, SDTP participants reporting past 12-month heroin or other opioid use during administration of the TCU Drug Screen II at prison intake and released to three Pennsylvania counties covered by the Recovery-Advocacy-Service-Empowerment (RASE) non-profit community recovery organization, were eligible for recruitment into the CRS program. RASE provides recovery support services in central Pennsylvania. Their philosophy is to employ a recovery-oriented system that is client-directed and support-centered. RASE CRSs identify and utilize client strengths and intentionally integrate their existing social support (e.g., friends, family, and other support groups) with professional assistance. Their services are delivered in a client’s natural environment where healing and relearning can be maximized. The RASE’s established infrastructure permitted the hiring of a pilot-specific CRS as well as the implementation of a CRS program to support SDTP participants in the transition from close supervision to community-based treatment. In Pennsylvania, a person in recovery can apply for a CRS credential with the state certification board if the person has a high school diploma, is in recovery for at least 2 years, completes a 54-hr training course, and passes the board examination. The pilot CRS was hired locally and was expected to act as a transition facilitator and a role model, sharing personal experiences of navigating life, recovery, and reentry with participants.

The peer support intervention proceeded in several stages, with the CRS first meeting pilot SDTP participants in inpatient treatment facilities to establish rapport, outline a recovery plan, and schedule an in-person meeting within 24 hr of release to community. Upon a participant’s consent, the CRS then made contact with a primary caregiver, usually a family member, to educate them on naloxone and its administration, along with providing a naloxone dose. The CRS also informed the caregiver of community-based substance use treatment programming and emergency care and help them facilitate their loved one’s reentry and recovery as needed. During the first post-release meeting, the CRSs provided participants a detailed and individualized recovery plan, based on individualized needs, covering domains such as lifestyle and relapse prevention, housing, employment, education, medical and mental health, and family and childcare. On average, the CRS then met or contacted each participant twice per week in the first month following release. The CRS also kept in touch with the caregivers to ensure that any additional concerns or comments from them were addressed in time.

### Sample

The Pennsylvania Department of Corrections, local inpatient treatment facilities, RASE personnel, and researchers worked together to identify and recruit eligible SDTP participants. This multi-agency collaboration was particularly crucial because the pilot was launched in the midst of the COVID-19 pandemic. Adjustments were made to comply with social distancing and quarantining per Centers for Disease Control (CDC)’s guidelines, including online recruitment, participant-CRS initial meeting in open space, and masking for in-person services.

Following University Institutional Review Board (IRB) and Pennsylvania Department of Corrections (PADOC) ethics approvals, implementation of the pilot program began in November of 2020. After 1 year of recruitment, fifteen reentrants and nine family members were recruited to receive the intervention. Of these, two reentrants opted out of the study prior to the one-month follow-up interview and two were removed due to reincarceration. This results in a retention rate of 73%, and a final sample of nine reentrants and four caregivers interviewed 1 month following custodial release. Of the nine interviewed reentrants, three were women (six men), two were Black (seven White), and they ranged in age from 21 to 51 years old. Prior to release from inpatient treatment, participants provided (via Zoom interview due to COVID-19 restrictions) informed consent, capacity to consent (University of California, San Diego Brief Assessment of Capacity to Consent [UBACC], Jeste et al., 2007), a substance use survey (TCU Drug Screen 5 + Opioid Supplement) and contact information for a primary caregiver. On average, participants reported high levels of substance use in the 12 months prior to incarceration, with (a) all participants reporting non-prescription opioid use, along with poly-substance use (e.g., methamphetamine, cocaine, alcohol, or marijuana) (b) seven participants reporting “extremely serious” drug problems, (c) five participants reporting daily heroin injection, and (d) six participants reporting at least one opioid overdose. Three participating caregivers were mothers, and the other was a female sibling. Pursuant to PADOC policy, SDTP participants were ineligible to receive any compensation. However, family members were provided a $30 gift certificate for their interview participation.

### Analytical Strategy

Telephone interviews were conducted with reentrants and their caregivers separately 1 month after the intervention. On average, an interview lasted 1 hr and covered various topics about the reentry and recovery process. Interviews were audio recorded and, with redacted personal information, transcribed by the Datagain secure transcription service for qualitative data analysis. In this study, we focused on 152 narrative elements (i.e., response subsections with a shared topic) directly related to the CRS component, elicited from questions about interviewees’ perceptions of the CRS and related services.

We employed iterative thematic coding strategies for the analysis (Braun & Clarke, 2006). Specifically, one coder conducted the initial open coding of a subsample of 40 narrative elements to identify general themes. Three additional coders then independently applied the themes to the same 40 narrative elements to ensure theme saturation and consistency (defined as the same themes identified by at least two coders for each narrative). Fourteen themes emerged in this initial coding process and were applied to all 152 CRS narrative elements from the interview transcripts. After coding all narratives, the original 14 themes were further revised and collapsed into ten themes.

## Results

Table 1 presents ten themes describing participants’ perceptions of CRS services in the order of their prevalence. Below, we summarize broad patterns in the data using direct quotes from reentrants and their family members (pseudonyms were created to maintain confidentiality).

**Table 1.** CRS Service-Related Themes Identified by Reentrants (*n* = 9) and Caregivers (*n* = 4).

Rank	Themes^a^	Prevalence^b^
1	Establishing services	30%
	a. Establishing health care & treatment (13%)	
	b. Establishing employment or financial services (13%)	
	c. Establishing other or unspecified services (10%)	
	d. Establishing Housing (9%)	
2	Ease transition/overall advocacy (non-specific)	24%
3	Guidance on Decisions	20%
4	Always available & checking in	19%
5	Emotional support	14%
5	Knowledge	14%
7	Coordinating with family	11%
7	Transportation	11%
9	Unnecessary/did not receive services	7%
10	Relatable	5%

a Fourteen narratives (9%) were unable to be placed in a theme (i.e., uncategorized).

b Prevalence is calculated as percentage of 152 narrative elements.

### Most Returning Participants and Their Caregivers Described the CRS Program in Highly Positive Terms and as Helpful for Reentrant Recovery

Ben: “The CRS has been wonderful. He’s helping me get a place, get a room in a Sober house. He takes me back and forth to my doctors. Whatever I need, he helps me with it. I’m doing really well. I mean, I’m happy for once.”

Pete: “I feel like this program is a necessity and I hope that more people have access to it. I hope it’s not just [SDTP]. I hope it’s anybody with drug related issues.”

Gordon: “I could not have done this without [the CRS], I couldn't be able to build myself the way that I have. It’s my [CRS] who helps me a lot. I recommend a [CRS] for anybody who wants to change, coming out of prison or a facility like a rehab center.”

Indeed, one client was so impacted by the CRS program that she enrolled in the CRS certification course herself.

Valerie: “The ultimate goal is to get paid [to help others maintain sobriety]. [The CRS] and I talk about it all the time. He’s like, ‘the DOC is now paying my salary because I didn’t [know] what I was supposed to do and now I want to help others.’ It’s surreal and the fact that that opportunity is there just puts it full throttle.”

### The Most Prevalent Theme Was That the CRS Helped Make Connections to Local Services

As expected, individuals discharged from inpatient treatment were in immediate need of community services, but navigating these services is potentially confusing for those exiting close custody confinement. The pilot CRS was able to use their local knowledge and experiences to make these connections. Reentrants and their caregivers particularly pointed to the CRS helping to establish health care and treatment services necessary for recovery.

Valerie: “I just feel like using [the CRS] and their services can weed through all of that stress and heartache. They can take the time to see what I want, what they feel is most beneficial, and help me get there.”

Pete: “I got out here [released to the community,] and I was totally lost to what they [the system] expected from me – how I was supposed to go about outpatient, all of that stuff. [The] CRS actually pretty much did all the work for me. If [the] CRS would have not helped me with half of this stuff, I don’t know what I would’ve already done, to be honest with you. I probably wouldn’t be as good as I am. I feel really solid and secure right now. . . CRS set me up with pain management doctors to work with injections, and he got me a personal care provider, and he took me to my appointments – it was a whole handful of stuff that I wasn’t ready to deal with. . . He has been introducing me to this NA/AA type stuff, which I’ve never been involved with.”

Additionally, some participants mentioned that their CRS encouraged them to seek employment opportunities and connect them with proper financial resources. Because of their lived experience, the CRS may be more able to build trust and convince participants to seek employment than a parole agent.

Valerie: “It [taking a course] is probably going to take me a little longer, which is why we [CRS and I] started discussing [a job]. [The CRS said,] ‘hey, why don’t we just get a part-time job in the meantime while you get the certification?’”

Valerie’s mother: “CRS said, ‘you know, maybe Valerie could work one day a week. I don’t know, I’m gonna talk to her about it.’ So, they talk every day. . . CRS said [to Valerie], ‘I’m not gonna put you in a warehouse and I’m not gonna put you around any food, because it’s easy to have cash. . . pocket it or whatever.’ So that made me happy, too.”

Several respondents mentioned the lack of financial resources to secure safe housing and that the CRS was able to connect them to resources that fund housing for reentering individuals.

Natalia: “I got funding for my rent, which everything is included, [and] the CRS worker told me about how to get that.”

Gloria: “Oh, what really made me happy [since being released] was my CRS worker was able to give me ties to people who can help me with funding for housing. That was amazing. That was a really great day that he gave me the resources for that. I was waiting for that in [a month]. That saves me a lot of stress and a lot of anxiety. . .I don’t have to go back out there and do illegal things, become explosive, and be a criminal again; like these people were able to help me and my CRS worker was able to give me that pool to connect me with those people.”

The CRS was also able to connect reentrants to other resources as situations arose.

Natalia: “I don’t have income right now. My friend has been helping me like if I need soap or something, but my CRS worker helped me apply for food stamp and stuff. . . He helped me get the application, my ID, and set me up with a bus pass. He offered to give me a free bus pass. He got me funding for months of free rent.”

Pete: “He [CRS] gave me food. When you first get to the halfway house, they don’t provide food, and there’s no assistance or anything. . .some of my roommates wouldn’t have eaten either. . .He even went to the food bank for us, too, on a Sunday, on his day off.”

CRS helping with establishing services was closely related to their local knowledge about services and reentry needs (Theme 5_(ii)_).

Pete: “Honestly, I moved to [another city] but I’m hoping not to lose him as my CRS. He’s just so helpful. He’s knowledgeable. If I’ve got a question about something or an issue about something, he’s got answers. If I’m like, look, bro, this is how I’m feeling about the situation and I don’t know what to deal with it, he’ll give me an idea or he’ll give me his thoughts on how he would handle it. Most of the time, it’s very helpful.”

Gloria: “He was showing me [the city] because he is very familiar with it. He was telling me what was good and what’s not good about [the city], like where the decent areas are and to help me make home plan out.”

Valerie: “He said he wants to come down there with me, advocate for me, say anything he can to help me with – he told me about this federal bonding program that’s in place to utilize. I never knew any of these things existed. He says it’s his job to act as that liaison, tell me everything that’s out there. I’m just soaking it all up.”

### Reentrants and Caregivers Were Thankful That the CRS Was an Advocate, “Had Their Backs,” and Helped With Decision-Making

Reentrants expressed gratitude for the general help and advocacy provided by their CRS that eased the transition from inpatient centers to the community. Positive words, such as “great,” “awesome,” “wonderful,” or “above and beyond,” were plentiful in interviewees’ overall impressions about the pilot CRS.

Gloria: “From what I’ve gotten out of it [the CRS services], it has been very beneficial. He [CRS] will advocate for me. Let’s say I was to slip up and got high or something like that, I went to my CRS worker, and he would sit there and advocate for me and help me get to a rehab, get to proper places where I need to be so I could survive. He’s definitely a big advocator. He’s in consistent contact with my counselor. I know they are in contact a lot and to see what’s going on with me, to make sure I’m okay.”

Pete: “It’s been hectic, but honestly, having [the CRS] has been a lifesaver. He has truly helped me. He helped me with my insurance through welfare. He helped me get things in order. He helped me get my medications. A lot of hurdles were a lot easier to overcome because he had my back.”

Having someone to consult, especially a CRS who can share personal experience of how they have made difficult decisions in similar situations, can sometimes guide the reentering individuals to staying on the right path. The pilot CRS giving good advice to the participants is another prevalent theme.

Nathaniel: “He already helped me with my food stamps. It’s not like he’s doing those things for me. He’s just pushing me in the right direction. . .He’s doing everything he can to help me.”

Gordon: “. . .CRS is out there in the world right now for somebody who is actually trying to change themselves. . . I know my CRS, I know that he has helped me with advice. . . strategizing this and strategizing that. . . I can’t explain how they could help me [in the future], but I know that if I ever needed anything, any advice, or needed [someone] to point in the right direction for anything, that would be the resource that I would go to.”

Valerie: “The very first day we [my CRS and I] made a list of my short-, mid-, and long-term goals. We did a recovery capital scale. He was able to get a full assessment of me and what my main areas are that he could help me. . . Him breaking down these goals and putting measurable outcomes to them and then every time I go in there seeing them, the numbers go up or me getting closer to the goals. Just writing them down in black and white, a simple exercise like that is just helping me visualize it, track my progress, see it.”

### Perceptions That the CRS Was Always Available and Checking to See How Participants Were Doing During the First Weeks of Reentry Were Commonly Mentioned as Positive Aspects of This CRS Program

Ben’s sister: “Ben just said that pretty much the CRS is always there if he needs to talk, if he has a problem. He said that the CRS has made it every known that if he feels like there’s something he feels he needs in his recovery in order to succeed, the CRS will try and help him to obtain it or try and help him work out a way to figure out how to obtain it.”

Knowing that the CRS is just a phone call away can be a great relief.

Pete: “He was actually straight across the street from that halfway house. The RASE Project’s office is right across the street, which is great because I needed it to be right across the street. Trust me. There were days that I would text him like, ‘Man, I really do have to get out of here.’ He would say, “okay, you alright?. . . I can meet you at this time if you need to talk [in person] or if you want to just talk on the phone.’ A lot of times, that really helped me out.”

Gloria: “He hits me up [i.e., calling] and I let him know what’s going on, mainly about [a personal issue] because I go off about that – it is the number one thing happening in my life. He tells me like everything’s going to be okay. He lets me know that everything’s going to be fine. He always checks in, even when I’m not make it there, he at least makes a point checking on me. He makes sure everything is fine with me. So he is an amazing person. He’s very nice, he’s hilarious, he has a good sense of humor and he’s a good guy. He really is. He does a good job. He does.”

Part of the availability of the CRS was that participants could easily get transportation to services and treatment.

Nathaniel: “He gives me rides. He told me if I need a ride to any doctor’s appointment, like that there are services out there that he can help, he said he’ll do it, or he’ll find somebody do it for me.”

Pete: “CRS actually came on his time off. He wasn’t even on the clock. He just wanted to make sure that I was alright, and he came and picked me up there one night. ‘Hey, I didn’t want you to walk back through the city in the freezing cold.’ I was impressed. I couldn’t even believe he did it. . . I had to change all plans and go to a place that was further away from home. . .so to change locations to [time length] away is a huge difference in transportation, and I have everything for my medical stuff set up for me here in [this city]. I have my doctors here, my surgeons here, and to get to all those appointments, had CRS not taken me, I would’ve never gotten to any of those appointments.”

Valerie’s mother: “So I talked to the CRS yesterday for 45 minutes and we talk, you know, every week and he said how well she’s going and doing everything. He got her a one-year [bus] coin and she was so thrilled with that coin.”

### CRS Effectiveness Also Included the Provision of Emotional Support, Caring, and Empathy

Participants stated that having someone to talk to, even briefly, made them feel supported during the precarious reentry period.

Ben’s sister: “Any other time, I feel like Ben’s answer would have been like, ‘you know what? Screw it. It doesn’t matter that they are railroading me, I’ll just go get high.’ But this time, he knew he was in the right and he had somebody to call. . . Instead of going to getting drugs, where I am sure he still knows a million people he could have called, he called the CRS. Because the CRS told him, ‘If you want to succeed and you show me you can succeed, I’ll help you succeed.’ And that’s what he did. He didn’t call the wrong number for a change, he called the right one. [laugh]”

Gloria: “He has helped [since I’ve been out]. And he’s been somebody I was able to vent to. And like I said, he’s helping me with the resources that I can’t get here, so he’s definitely been a good help. And he checks in on me a lot. . . He’s the one that if I feel like I need to use [drug], he’s there. He’s somebody I can come and talk to and he could talk me out of that. So that’s a good thing.”

Perceptions of CRS emotional support parallel perceptions that the CRS was relatable and easy to talk to (Theme 10).

Nathaniel: “I mean, the CRS is chill. He is pretty cool. He’s somebody I can relate to. . . As far as I can see right now what CRS and RASE are doing for me, they go above and beyond what they could do. That’s the way I feel about it and I’m pretty sure lot has to do with CRS. . . I just feel as though I can relate to him and do better.”

### CRS Connections With Caregivers Were Perceived as Important for Recovery

Most interviewees, participants as well as caregivers, provided positive comments on the CRS-family connection, which is a novel component of this specific pilot.

Pete: “I think him [CRS] talking to my family like letting my brother know what I’m involved in and how I’m trying to change and that he is there to help, I think it’s given my family a little bit of a security. They feel like if something is wrong, they have somebody that is going to let them know because, obviously, I didn’t let them know when something went wrong last time, so I think they rely on him to, in a sense, keep an eye on me.”

Ben’s sister: “Ben told him [CRS] that he really felt that getting to see us was something that he needed. . .and CRS said, ‘well, then let’s figure out how to make this happen, let’s figure out how to get you there.’ I guess CRS encouraged him to keep trying to get in touch with me.”

Valerie: “The fact that he has reached out to my mother, I’m sure that’s part of this where you maybe contact, but I know he’s gone above and beyond with her just from the conversations I’ve had with her. It’s another thing that I’ve never been offered or that’s been in place for me post any kind of release. . . he’s always made himself available to me, always, to me, myself, my mother, my counselor if they need to talk back and forth.”

Valerie’s mother: “He [CRS] said, ‘I’m not a PO [parole officer]. I’m a person to help her [Valerie] and guide her through like what she needs to do and to help her because we believe 100% in her.’ And that made my heart swell, so. The first time we talked, it was an hour and a half, so it’s good. I mean I’ve never had anybody reach out before to me to talk about Valerie ever.”

We also learned that not all reentrants maintained a good relationship with their family members, and not all caregivers wanted engagement with the CRS. For instance, one participant in our pilot decided not to introduce the CRS to their family, not because of a bad familial relationship but because the participant was dealing with issues in which family involvement would induce more stress and thus hinder reentry progress. Only when both a participant and their family members perceive potential benefit of engagement during reentry and recovery can the CRS-family connection be positive.

### Not All Participants Felt That They Needed a CRS, Either Because They Felt They Could Handle Their Own Problems or Believed That CRS Support Was Unnecessary

David: “I barely talked to CRS. Probably [I would talk to CRS about the desire to use or to sell drugs], I mean like, maybe sometimes. It depends. Probably not. I don’t know. It’s been maybe and maybe not. Like it depends on how I feel— I really haven’t had no urges.”

Jason: “I don’t know what they have to offer me that I need [in the future]. I’m already with another program for housing. . . No, I really don’t want anything – I mean, not more than anybody else right now.”

It should be noted, however, that these two reentrants (and one caregiver) were recruited close to the end of the pilot period, leading to insufficient exposure to the CRS services. It is likely that their opinions would alter and become similar to the other interviewees had they had a few more weeks to interact with the CRS. This suggests that CRS effectiveness depends on continued CRS contact and the maintenance of a high level of individualized care, which may be difficult over extended periods but could be effective and feasible during the first few weeks of transition.

## Discussion

This CRS pilot study targeted the transition from close custody confinement to community supervision when risks of relapse and rearrest are extremely high. The interview transcript analysis showed that CRS assistance was overwhelmingly perceived as positive by study participants and their caregivers. Results suggest that CRSs are in a unique position of gaining the trust of SUD reentrants and can be essential for building recovery capital during the reentry transition. Local knowledge, lived experience, advocacy, availability, empathy, and family outreach are identified as key mechanisms of CRS success distinctive from other post-release programs.

Results from this pilot program, consistent with the limited number of existing studies (Barrenger et al., 2020; Cos et al., 2020; Ray et al., 2021; Reingle Gonzalez et al., 2019; Tracy et al., 2011), contribute to our knowledge of a recently emerged phenomenon in the criminal justice context, demonstrating that the incorporation of CRS services in the critical period of reentry transition is not only feasible but also recommended for reentrants. This pilot study also shows that connecting family members to a CRS enhances the reentrant’s recovery capital, (re)builds social bonds in an important life course transition (Hirschi, 1969; Sampson & Laub, 1993), and should be considered as an added value for increasing reentry and recovery success.

The prosperity of a CRS program is not one-sided: CRSs themselves also benefit from assisting others’ reentry and recovery. Scholars have suggested that CRSs perceive their peer-supporting role as highly rewarding and satisfying, contributing to their own recovery process (Reingle Gonzalez et al., 2019; Whyte, 2011). LeBel (2007) examined the benefits of helping others for formerly incarcerated individuals and found that the “helper/wounded healer” orientation is associated with increased life satisfaction and reduced criminality and likelihood of rearrest. While the experience of CRSs is not a focus of the current study, this “helper” orientation and its related fulfillment of helping others were certainly shared by the pilot CRSs throughout the research collaboration. The CRS position provides a “turning point” (Sampson & Laub, 1993) for shifting self-perception from a negative, stigma-oriented, formerly justice-involved image to a positive, mentoring-oriented, helper portrait. Indeed, the pilot CRS in this study was perceived as extremely knowledgeable and helpful, so much so that one returning individual was inspired and taking serious actions to become a CRS herself. Such positive change in the mindset of reentrants, especially seeded prior to and early during reentry, can no doubt bring them hope and empowerment for the pursuit of a meaningful life after the departure from prison.

This pilot program and study results are subject to limitations. The COVID-19 pandemic substantially impacted recruitment, which was originally aimed at 30 participants. Because this was a small-sample pilot program with only one community-based recovery provider and highly motivated CRSs, we are also unable to make causal claims and generalizations about the intervention’s impacts on objective measures of relapse or recidivism. It is possible that positive intervention effects would be lessened, if not disappearing altogether, with a larger sample, multiple recovery organizations, and many CRSs, particularly if the sample extends beyond SDTP participants with opioid use disorders to include general population reentrants. Estimation of causal effects of a CRS intervention requires a larger and more diverse sample, more CRSs and recovery organizations, quantitative data on relapse, rearrest, and reincarceration, and ideally a randomized controlled trial (Bassuk et al., 2016; Eddie et al., 2019; Tolan et al., 2013). Additionally, implementing a similar program at scale would include many CRSs to estimate and understand average treatment effects, which are essential for long-term sustainability.

Nonetheless, the pilot created an interorganizational infrastructure (with corrections agencies, community service providers, and families) to implement a CRS program for reentrants with SUD. This generates insight that is useful for any attempt to implement a CRS intervention for prison or jail reentrants in recovery. Importantly, findings from this pilot program provide best practices and essential program ingredients that can incorporated into a training curriculum for CRSs providing reentrant care. At the same time, the CRS model is not a one-size-fits-all approach, so any CRS training cannot be boiled down to a specific list of activities and practices. Instead, it seems to thrive on the personal rapport between a CRS and a recovering peer and the ability of the CRS to tune into, and be responsive to, the emergent needs of the peer during the reentry transition. This bonding over shared experiences separates CRSs from corrections agents, counselors, and medical professionals. Our study suggests that it is important to initiate this CRS-peer rapport prior to release; delaying until reentrants are already in the community creates missed opportunities to engage potential peers in services and support which they and their families may need.
